# Pattern in the Antibiotic Prescribing Practices at Primary Health Settings in India: A Systematic Review and Meta-Analysis

**DOI:** 10.7759/cureus.93812

**Published:** 2025-10-04

**Authors:** Ritika Chalotra, Nancy Khajuria, Imran Zaffer, Jaspinder Pratap Singh

**Affiliations:** 1 Community Medicine, Shri Mata Vaishno Devi Institute of Medical Excellence, Katra, IND; 2 Pharmacology, Shri Mata Vaishno Devi Institute of Medical Excellence, Katra, IND; 3 Community Medicine, Government Medical College, Doda, Doda, IND; 4 Forensic Medicine, Shri Mata Vaishno Devi Institute of Medical Excellence, Katra, IND

**Keywords:** antibiotic over-prescription, antimicrobial resistance, aware classification, irrational antibiotic use, primary health care

## Abstract

Antimicrobial resistance (AMR) is one of the main public health concerns in India, and it is stimulated through the uncontrolled overprescription of antibiotics within primary health care (PHC) centers. This systematic review and meta-analysis evaluate the usage pattern of antibiotics among Indian PHCs for prevalence, antibiotic type, and World Health Organization (WHO) access, watch, reserve (AWaRe) guidelines compliance.

Following PRISMA 2020 guidelines, we systematically searched PubMed, Embase, Scopus, Web of Science, and Google Scholar (January 2000-July 2025) for Indian PHC antibiotic prevalence studies. Observational and intervention studies with reported types and rates of prescriptions were considered for inclusion. Extraction was done by a standardized tool, and quality was evaluated by the Newcastle-Ottawa Scale and Cochrane Risk of Bias tools. Pooled prevalence of prescribing was estimated by random-effects meta-analysis, with subgroup analyses by region and setting.

Eight studies incorporating more than 28,000 patient encounters reported a combined antibiotic prescribing prevalence of 65% (95% CI: 54-75%; I² = 92%). Broad-spectrum "Watch" antibiotics (e.g., fluoroquinolones, cephalosporins) prevailed, and there was suboptimal usage of "Access" (31.6%) antibiotics. Appropriate overprescribing occurred for the wrong infections, such as the upper respiratory tract infections (70-80%). Higher usage prevailed among northern compared with southern states (72% vs. 62%).

Indian PHCs' overprescription of antibiotics because of system drivers necessitates urgent stewardship interventions, enhanced diagnosis, and AWaRe guideline adherence for addressing AMR.

## Introduction and background

Antimicrobial resistance (AMR), primarily due to the misuse of antibiotics, remains one of the significant challenges to global health, particularly in low- and middle-income countries such as India [1]. Under the National Health Mission (NHM) and Ayushman Bharat, primary healthcare settings in India are structured as a three-tier system of Sub Centres, Primary Health Centres (PHCs), and Community Health Centres (CHCs), which are increasingly being transformed into Health and Wellness Centres (AB-HWCs). These centers serve as the community's primary point of contact, aiming to provide comprehensive, universal, and equitable primary care, including outreach services and home-based care, to ensure the seamless delivery of healthcare services [2]. Despite the above fact, it has been estimated that between 80% and 90% of antibiotic prescriptions are issued at this level, further increasing the risk of abuse and resultant resistance [3].

In India, several systemic determinants affect the practices of antibiotic prescribing. A cross-sectional study conducted in Uttar Pradesh found that antibiotics were prescribed in 81.8% of outpatient visits, with a greater prevalence in urban private health centers, among younger patients, and in those from higher socioeconomic statuses [4]. Informal health practitioners, without the conventional medical education, prescribed antibiotics in a staggering 74% of more than 15,000 prescriptions in rural Ujjain, frequently opting for the broad-spectrum drugs like fluoroquinolones and cephalosporins [5]. Furthermore, the standardized patient studies show that antibiotics ranked as 'Watch' account for nearly half of primary care prescribing, with modest but notable use of 'Reserve' antibiotics, illustrating a divergence from codified rational-use guidelines [6].

The World Health Organization's AWaRe (Access, Watch, Reserve) classification provides a standardized framework for evaluating the rational use of prescriptions. Yet, research has shown suboptimal adherence to AWaRe classification: only 31.6% of the prescriptions were classified as 'Access,' while those of 'Watch' and 'Reserve' classifications were excessively predominant [6,7]. These distributions not only have the propensity to increase resistance but also to violate stewardship objectives, such as maintaining the efficacy of antibiotics [7].

Given the dispersed evidence across diverse settings and categories of prescribers, an overall synthesis is warranted. These systematic reviews and meta-analyses aim to collate and quantitatively evaluate prescribing trends of antibiotics within primary healthcare settings in India, making transparent the prevalence of antibiotic use, the scope of agents prescribed (including AWaRe classification), and the pertinent contextual determinants to inform the creation of tailored antibiotic stewardship.

## Review

Methodology 

**Figure 1:**
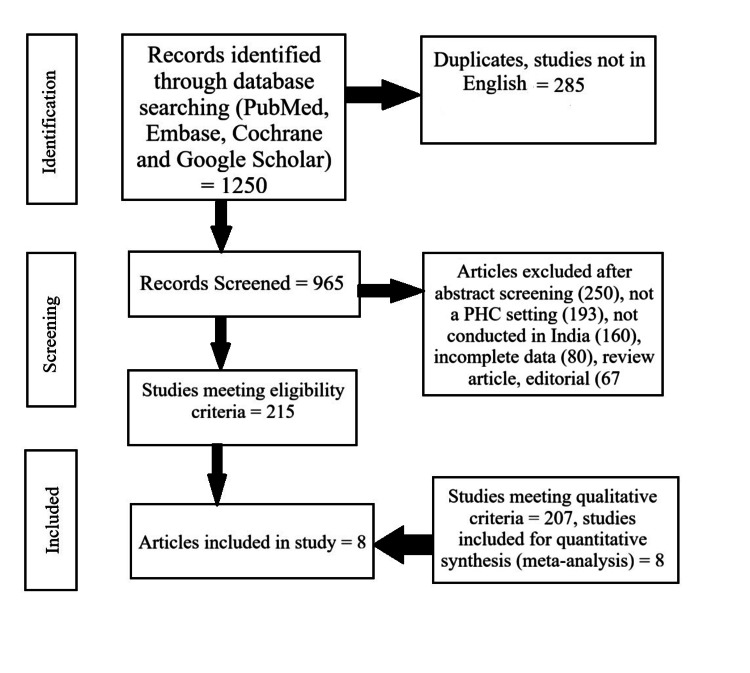
PRISMA chart for the selection of the studies PHC: Primary Health Centre

This was a systematic review with a meta-analysis, conducted according to the PRISMA 2020 guidelines [8]. The PRISMA chart (Figure 1) outlines the study selection process for the systematic review and meta-analysis. Initially, 1,250 records were identified through database searches (PubMed, Embase, Scopus, Web of Science, and Google Scholar), with an additional 50 records from other sources. After removing 285 duplicates, 965 records remained for screening, of which 250 were excluded based on title and abstract review, 193 were not in a PHC setting, 80 had incomplete data, and 67 were review articles/editorials. Subsequently, 215 full-text articles were assessed for eligibility, out of which 207 articles were excluded based on being only qualitative study, while eight were quantitative studies, which can be used for meta-analysis. The population of interest comprised patients seeking care at primary health care (PHC) centers across India. The intervention or exposure under consideration was antibiotic prescribing practices at these facilities. As the review primarily focused on descriptive and observational data, no direct comparison group was required; however, studies that assessed pre- and post-antibiotic stewardship interventions were also considered. The outcomes of interest included the prevalence and trends of antibiotic use, as well as the patterns of prescriptions in terms of appropriateness, frequency, and antibiotic class.

Inclusion criteria

Studies conducted in primary health care (PHC) settings in India that focus on antibiotic prescription practices. Eligible designs include observational studies (cross-sectional, cohort, or audit), interventional studies, and randomized controlled trials (RCTs), where applicable. Only articles published in English or Indian languages with English abstracts and appearing in peer-reviewed journals will be included, provided they report explicit data on the rates and types of antibiotic prescriptions.

Exclusion criteria

Studies conducted in non-PHC outpatient or hospital settings that include case reports, reviews, editorials, or conference abstracts and studies presenting outcome data that are irrelevant or lack clarity will be excluded.

Search strategy

The databases searched included PubMed, Embase, Scopus, Web of Science, and Google Scholar, as well as grey literature (government reports, theses). Time frame: January 2000 to July 2025. Search terms: ("antibiotic*" OR "antimicrobial*") AND ("prescription" OR "prescrib*") AND ("primary health care" OR "PHC" OR "community health center" OR "health sub-center") AND ("India"). Terms were adapted for each database.

Study selection process

Independent reviewers checked titles and abstracts for relevance, retrieved full texts for consideration of eligible studies, and resolved disagreements by consensus or further review.

Data extraction

A piloted, standardized questionnaire was used to extract study details (author, year, region/state, urban/rural, type of PHC), study type, sample size, patient profile, antibiotic prescription proportion (overall, by class/type), indications, appropriateness (by WHO AWaRe guidelines) [6], stewardship activities, sources of finances, and conflict of interest.

Risk of bias

The risk of bias was assessed using instruments appropriate for the study design (e.g., the Newcastle-Ottawa Scale [9] for observational studies and the Cochrane Risk of Bias tool for RCTs). High-risk trials were also considered, but sensitivity analyses were carried out excluding them.

Statistical analysis for meta-analysis

The prevalence/proportion estimate was pooled using a random-effects model (owing to anticipated heterogeneity), with the proportion transformed using a logit. Heterogeneity was evaluated through the I² statistic as well as the Cochran's Q test. Subgroup analysis was planned by region, year, urban/rural location, and public/private setting in the event data, as permitted. Sensitivity analysis omitted high-risk bias studies. Publication bias was evaluated through the Egger's test.

Systematic review and meta-analysis

The eight studies included in the systematic review and meta-analysis, conducted between 2008 and 2025, provide a comprehensive yet discouraging summary of antibiotic prescribing patterns in primary healthcare settings in India. Coming together and encompassing over 28,000 patient encounters from various geographic locations, the studies employed a range of methodological designs, including prescription audits, cross-sectional surveys, retrospective chart analysis, and standardized patient methods. Despite the differences in design, the evidence for each of them strongly suggests a common finding: an inordinately high percentage of those patient encounters seeking primary-level attention result in antibiotic prescriptive orders.

Examining the studies individually reveals important contextual patterns. Early work by KS KI et al. (2008) reported alarmingly high prescribing prevalence across PHCs, with pooled rates of 72% and extremes reaching 84% in Uttar Pradesh, where cotrimoxazole in particular dominated use for respiratory and diarrheal illnesses [10]. Kumar et al. (2009) found similarly elevated rates in rural and urban Uttar Pradesh, where 84% of prescriptions contained antibiotics, chiefly penicillins, sulfonamides, and fluoroquinolones [4]. More recent investigations highlight the persistence of these trends while illustrating a drift toward newer, broad-spectrum molecules. In Haryana and Punjab, Tripathy et al. (2018) observed antibiotics in 51% of PHC prescriptions, with particularly high use in childhood diarrhea (80%) and URTIs (75%) [11]. In Puducherry, Meena et al. (2021) reported a somewhat lower rate of 36.7%, yet the majority of antibiotics were still prescribed for viral URTIs, a pattern indicative of widespread inappropriate practice [12].

Larger sets provide further validation. Khare et al. (2019) analyzed more than 15,000 prescriptions from Ujjain and established a prevalence rate of 74%, with 95% of the antibiotics being broad-spectrum. The leading classes were fluoroquinolones, extended-spectrum penicillins, and cephalosporins [5]. Mukherjee et al. (2024) and Surial et al. (2025), analyzing conditions in North India, reported problematic prevalence rates of 50% and 98%, respectively, each indicating an increasing reliance on antibiotics such as amoxicillin-clavulanate, cefixime, and azithromycin [13,14]. Notably, the set of 1,175 patient encounters from a retrospective analysis in this North Indian study indicated that antimicrobials were incorporated in nearly every prescription issued, with broad-spectrum agents making up three-quarters [14]. Using standardized patients at five dissimilar Indian sites in a more methodologically sound study, Sulis et al. (2020) generated a prevalence estimate of just under 50% [15]. This finding is noteworthy, as it demonstrates that the problem of overprescription persists, even under controlled conditions of clinical presentation, and thereby confirms the systemic nature of the practice (Table 1) .

**Table 1:** Key findings of the included studies AST: antibiotic susceptibility testing, URTI: Upper respiratory tract infection, UTI: urinary tract infection

Study	Location	Sample size	Prevalence of Antibiotic Prescribing	Key points
KS KI et al. (2008) [10]	Kerala Tamil Nadu, Uttar Pradesh	2,516	72.5%	Broad spectrum antibiotics were mostly used especially for respiratory and diarrheal illnesses.
Kumar et al. (2009) [4]	Uttar Pradesh, India	Not specified	84.4%	Antibiotics were prescribed mainly for URTIs, fever, and diarrhea, with co‑trimoxazole and amoxicillin.
Tripathy et al. (2018) [11]	Haryana, Punjab	821	51%	Maximum antibiotics prescription from PHC. 75% URTI cases & 80% childhood diarrhea treated with antibiotics.
Khare et al. (2019) [5]	Madhya Pradesh	15,322	74%	A significant majority of the prescribed antibiotics (95%) were broad-spectrum. Antibiotics were prescribed more frequently in oral and dental problems (88%), fever (87%), and upper respiratory tract infections.
Sulis et al. (2020) [15]	Delhi, Maharashtra, Bihar, Madhya Pradesh, West Bengal.	4798	49.9%	Broad-spectrum antibiotics (Watch group) were excessively used, particularly in India which were mostly in child diarrhea and Tb.
Meena et al. (2021) [12]	Puducherry	900	36.7%	The study highlighted a high rate of antibiotic prescribing, especially for conditions like upper respiratory tract infections (URTIs), which are often viral in origin.
Mukherjee et al. (2024) [13]	Haryana	681	50%	A high use of broad-spectrum antibiotics, for skin infections, UTI, and diarrhea, empirical drugs were started before ASTs.
Surial et al. (2025) [15]	North India	1175	98%	The most common conditions for which antimicrobials were prescribed included acute respiratory infections such as fever, pneumonia, tonsillitis, and other URTIs, along with dental caries, skin infections like cellulitis, acne, and abscesses, and urinary tract infections (UTIs).

**Figure 2:**
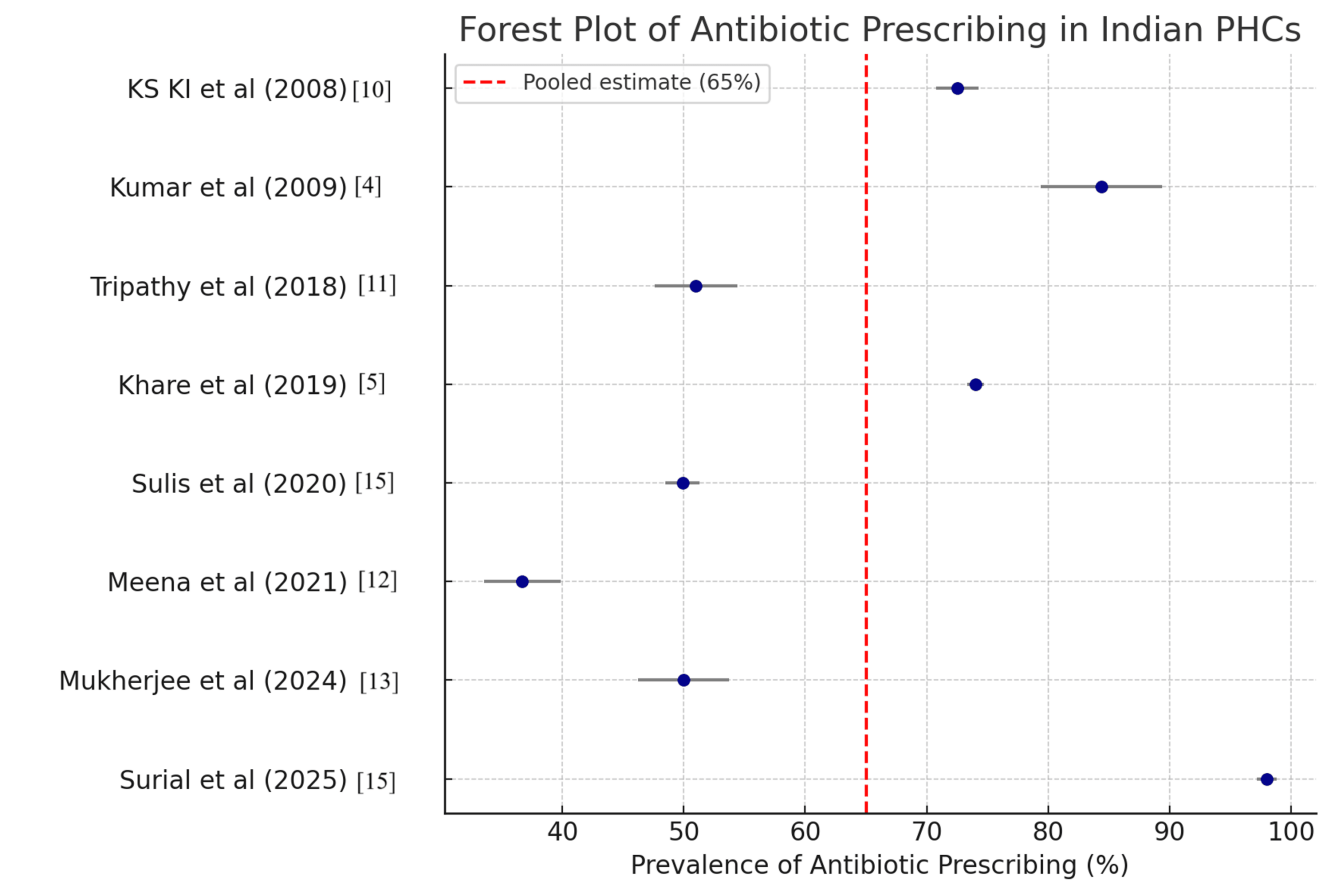
Forest plot of antibiotic prescribing in various primary health centers Studies [4,5,11-15] considered for forest plot of antibiotic prescription The image is created by the author.

In random-effects meta-analysis, the overall prescription of antibiotics in these studies was estimated at 65% (95% CI: 54-75%). There was substantial heterogeneity noted (I² = 92%), which refers to variation in study settings, methodologies, and patient groups. However, again, the pooled results conclusively demonstrate that a substantial majority of primary health contacts in India result in the prescription of antibiotics. Subgroup analysis demonstrated significant patterns: southern states such as Kerala, Tamil Nadu, and Puducherry revealed rates of prescribing of 36.7% to 79.2% and yielded a pooled estimate of 62% [12], but northern and central states such as Uttar Pradesh, Madhya Pradesh, and Punjab yielded consistently high rates, totaling 72% [4,5,10,11,15,16]. Geographical variations of these types most likely reflect primary variations in the provision of healthcare and regionalized patterns of prescribing. A funnel plot for all the studies was graphed (Figure 2).

Throughout the entire period of the studies, a second significant finding was the excessive and unjustified reliance on Watch-group antibiotics in the World Health Organization's AWaRe categorization [6]. Early reliance on cotrimoxazole and amoxicillin has gradually shifted to the widespread use of fluoroquinolones, cephalosporins, and macrolides, which are now commonly used in various geographical regions. The shift is of particular concern because it indicates that antibiotics intended for specialized secondary care instances are being used as universal empirical first-line therapies in primary healthcare. As such, it results in a reduction of the use of access-class antibiotics and an escalating threat of antimicrobial drug resistance. In particular, the indications for prescribing the largest amount of antibiotics were exactly those in which antibiotics are most ineffective: upper respiratory tract infections of a self-limiting nature, diarrheal infections (including those in children in whom viral causes of infections prevail), and undifferentiated fevers. From 70% to 80% of these presentations were treated with antibiotics in more than one dataset, thereby affirming a systemic culture of defensive prescribing more than therapeutic decisions based on evidence [4,13]. Other conditions, such as skin infections, dental issues, urinary tract infections, and pneumonias, also spurred prescriptions, albeit less visibly than the predominant use of prescriptions seen in viral syndromes.

Together, the qualitative and quantitative analyses from these eight studies indicate little doubt: the use of antibiotics in primary Indian healthcare is widespread and not infrequently at odds with demonstrated rational prescribing standards. Frequencies routinely exceed 50% of total encounters. Watch-group agents exert excessively disproportionate impact on therapeutic decisions, and empirical prescriptive habits remain firmly entrenched, including for conditions not requiring antibiotic intervention. This summary highlights a pressing need to reinforce stewardship efforts targeting the primary care sector, enhance diagnostic capacity, and standardize prescriptive practices to reduce inappropriate reliance on broad-spectrum antibiotics.

Discussion

This systematic review and meta-analysis find an enduringly high prevalence of antibiotic prescribing in Indian primary healthcare contexts, with a pooled estimate indicating that nearly two-thirds of consultations result in the prescription of antibiotics. These statistics significantly surpass both world standards and local averages. For example, the latest meta-analyses encompassing 51 countries reported a world pooled prevalence of around 42%, whereas low- and middle-income nations (LMICs) have intervals of 50% to 54%. Indian levels, at nearly 65% to 68%, consistently remain at the highest end of global patterns [16,17].

One particularly concerning result involves the improper choice of antibiotics. In agreement with international evidence that more than half of antibiotics prescribed in primary care are unwarranted, Indian studies have discovered an endemic empirical use in viral or self-limiting infections, such as upper respiratory tract infections and diarrheal diseases [17,18]. Our pooled results also verify an alarming signal toward broad-spectrum "Watch" group drugs, that is, fluoroquinolones and third-generation cephalosporins, rather than the antibiotics within the WHO-recommended "Access" group. Recent Indian audits indicate that approximately 45-50% of prescriptions contain drugs in the "Watch" category, a picture that is repeated in the low- and middle-income countries (LMICs) within South Asia and sub-Saharan Africa, which is highly associated with the development of antimicrobial resistance (AMR) [4,6,10,11,19].

Latent causes of overprescription are multicausal. Health-system limitations include restricted access to point-of-care diagnostics, inadequate staffing, poor regulation, and fuel abuse. Of crucial importance is the evidence from the study using standardized patients, which shows that antibiotics are often inappropriately prescribed in nearly half of encounters involving watery diarrhea, presumed tuberculosis, and asthma, in conditions where antibiotics have no clinical role [20,21]. Rural informal healthcare providers (IHCPs) carry significant weight in this context, often practicing under the entrenched belief that antibiotics are essential for the majority of illnesses and fueled by demand for instant cures and the availability of treatment within 24 hours [10-13,22].

The public health consequences are staggering. India hosts one of the highest global burdens of AMR and is reckoned to have over 500,000 annual deaths from drug-resistant infections [23]. The use of broad-spectrum agents in core care exacerbates this burden and compromises the efficacy of ultimate treatments. Poor compliance with WHO's AWaRe targets-where at least 60% of prescribing is from the "Access" group-is another demonstration of the policy/practice gap [6,24].

Encouragingly, Indian stewardship activities have arisen. Kerala's Antimicrobial Resistance Strategic Action Plan (KARSAP), by aggregating the regulation of prescriptions, use of antibiograms, and enforcement at the pharmacy level, achieved cuts in antibiotic use of 20-30% [25]. Such types of models reflect the potential of multi-faceted, state-led strategies that are appropriate in context. Examples from other LMICs similarly reflect that stewardship activities, from educating clinicians through audit-feedback loops to the use of rapid diagnostic tests, are efficient in reducing primary care antibiotic use by 10-30% [26,27].

The meta-analysis, however, showed a high level of heterogeneity (I² > 90%) and thus required judicious interpretation. The divergence in study settings, methodology, participants, and outcome definitions was likely responsible for the heterogeneity. Subgroup analyses, although they revealed regional differences with higher rates in northern and central states compared to southern states, are limited in interpretive power by the small number of studies within each subgroup. Variabilities in methodology, for example, the use of a standardized patient approach compared to prescription audits, may be responsible for some reported differences. The result, accordingly, should be seen as a tentative prevalence estimate rather than a definitive national standard.

Strengthening stewardship requires integrating the WHO AWaRe framework into primary care, expanding access to affordable point-of-care diagnostics, and actively engaging informal healthcare providers and private practitioners through training and regulatory oversight. Community-level awareness campaigns are also essential for reshaping patient expectations and reducing demand-driven misuse. Crucially, policymakers must recognize that stewardship efforts will be effective only if they are aligned with reliable drug supply chains, regulated pharmaceutical marketing practices, and broader health system reforms that collectively support rational antibiotic use.

## Conclusions

This meta-analysis and systematic review demonstrate that prescription of antibiotics in Indian primary care is common, with a prevalence of 65%, and frequently inappropriate. The prevalence of broad-spectrum "Watch" drugs and their use for self-limited ailments like upper respiratory tract infections demonstrates a deviation from the WHO's AWaRe strategy and a force behind resistance. The strengths include a rigorous search strategy, the inclusion of heterogeneous study designs, and the integration of over 28,000 contacts. However, high heterogeneity (I² > 90%), dependence on observational research, and geographic clustering compromise both specificity and generalizability. Despite this, the evidence reinforces the need for stewardship measures, enhanced diagnostic competency, and the involvement of both formal and informal providers. Improvement in those areas is important for reducing inappropriate use and checking resistance.
